# Residue 39 of Kir6.2 drives a difference in ATP sensitivity in human and canine beta-cell K_ATP_ channels

**DOI:** 10.3389/fphys.2025.1693112

**Published:** 2025-10-17

**Authors:** Natascia Vedovato, Frances M. Ashcroft, Brian Catchpole, Lucy J. Davison

**Affiliations:** ^1^ Department of Clinical Science and Services, The Royal Veterinary College, London, United Kingdom; ^2^ Department of Physiology, Anatomy and Genetics, University of Oxford, Oxford, United Kingdom; ^3^ Department of Pathobiology and Population Sciences, The Royal Veterinary College, London, United Kingdom

**Keywords:** K_ATP_ channels, ABCC8 and KCNJ11 genes, multi-species studies, pancreatic beta-cells, Kir6.2

## Abstract

ATP-sensitive potassium (K_ATP_) channels link beta-cell metabolism to electrical activity. By modulating the beta-cell membrane potential, they finely regulate glucose-stimulated insulin secretion. K_ATP_ channels are hetero-octameric complexes composed of four pore-forming subunits (Kir6.2, encoded by *KCNJ11*) and four regulatory subunits (SUR1, encoded by *ABCC8*). A multi-species alignment of the *KCNJ11* gene revealed that, although the sequence is highly conserved, residue 39 varies among different mammals. Previous studies have shown that this residue plays a critical role in regulating K_ATP_ channel activity and its mutation results in neonatal diabetes in humans. We therefore explored whether the canine and human K_ATP_ channel show different ATP sensitivities as a result of their sequence variation. We used patch-clamp electrophysiology to investigate species variation in the ATP sensitivity of the K_ATP_ channel. Functional studies showed that canine K_ATP_ channels exhibit reduced ATP sensitivity compared to human channels. However, stimulation by MgADP was unaffected. We next compared the ATP sensitivity of hybrid channels (human Kir6.2 with canine SUR1, and *vice versa*), as well as K_ATP_ channels in which residue 39 was swapped between human and canine Kir6.2. In each case, ATP sensitivity was mainly determined by the identity of the residue at position 39. Our study suggests that the ATP sensitivity of the pancreatic K_ATP_ channel differs between human and dog. This suggests that the beta-cell membrane potential and potentially insulin release may be fine-tuned differently across species.

## Graphical Abstract

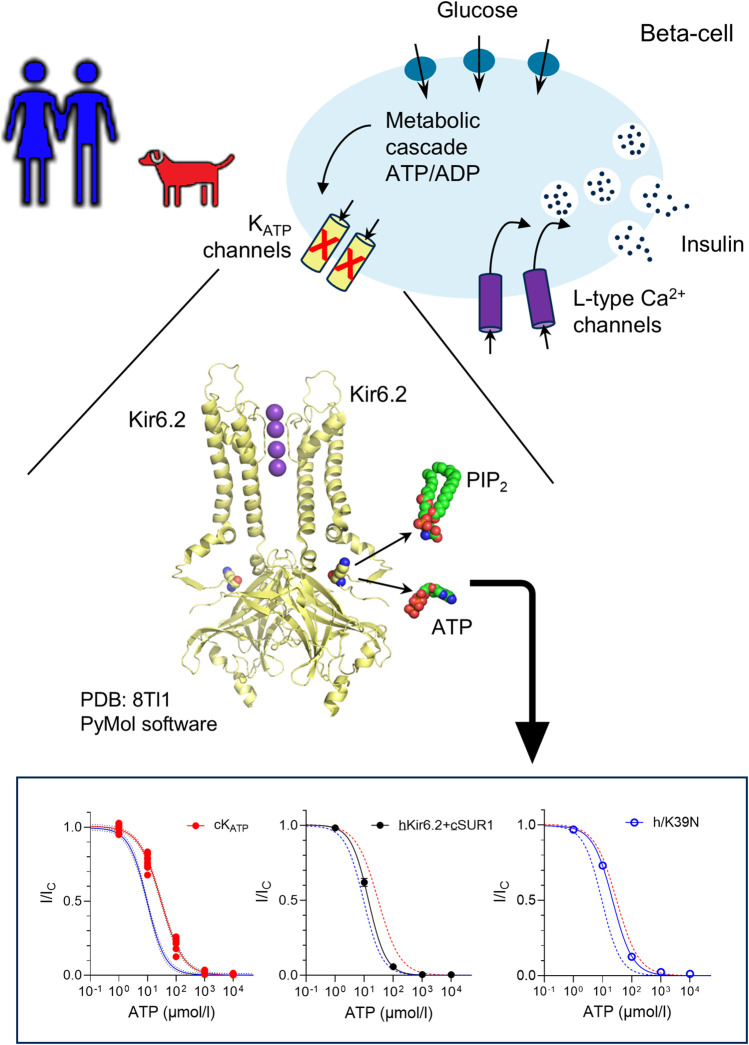

## 1 Introduction

Pancreatic beta-cells play a central role in maintaining glucose homeostasis by sensing blood glucose levels and secreting insulin accordingly (Rorsman and Ashcroft, 2017). Beta-cell biology is, in many aspects evolutionarily conserved, but significant species-specific differences exist (Tritschler et al., 2017). For example, different species express distinct types of beta-cell glucose importers and glucokinases (Heimberg et al., 1995), and normal blood glucose concentrations vary across species (Kahn and Line, 2005). These differences are expected, as species have evolved physiological adaptations to accommodate diverse diets, food availability, activity levels, and environmental factors. These physiological differences underscore the importance of understanding the molecular mechanisms that govern beta-cell function across species.

ATP-sensitive potassium (K_ATP_) channels serve as key metabolic sensors linking cellular energy status to electrical activity and insulin secretion (Rorsman and Ashcroft, 2017). Structurally, K_ATP_ channels are hetero-octameric complexes (reviewed in (Puljung, 2018; Driggers and Shyng, 2022)) composed of four inwardly rectifying potassium channel subunits (Kir6.2, encoded by the *KCNJ11* gene) and four regulatory sulfonylurea receptor one subunits (SUR1, encoded by *ABCC8*). Their activity is tightly regulated by a complex interplay of intracellular ligands that reflect the metabolic state of the cell. ATP binds to Kir6.2 and induces channel closure (Tucker et al., 1997), leading to membrane depolarization and insulin secretion. In contrast, phosphoinositides such as phosphatidylinositol 4,5-bisphosphate (PIP_2_) bind to a separate site on Kir6.2 and promote the open state of the channel, counteracting ATP-mediated inhibition (Baukrowitz et al., 1998; Shyng and Nichols, 1998; Driggers et al., 2024). Furthermore, MgADP/MgATP binds to the SUR1 subunit and simulates channel opening, thereby hyperpolarizing the membrane and suppressing insulin release (Gribble et al., 1997; Shyng et al., 1997). These opposing regulatory inputs, allow K_ATP_ channels to act as metabolic sensors, finely tuning beta-cell excitability.

Gain-of-function mutations and common variants in the human genes encoding the pore-forming *(KCNJ11)* and regulatory *(ABCC8)* subunits of the K_ATP_ channel have been implicated in neonatal diabetes (ND), early-onset diabetes, and an increased risk of developing type 2 diabetes later in life (Hattersley and Ashcroft, 2005; Pipatpolkai et al., 2020). Such mutations typically impair metabolic (ATP) inhibition of channel activity. Conversely, loss-of-function mutations produce hyperinsulinism and hypoglycaemia (Dunne et al., 2004; Cosgrove et al., 2004; De Franco et al., 2020). We hypothesized that differences in the sequence of Kir6.2 subunit between humans and non-human species, such as the dog, might also lead to changes in channel ATP sensitivity, with potential implications for glucose tolerance and diabetes risk across species.

## 2 Materials and methods

### 2.1 Molecular biology

Recombinant wild-type human or canine *KCNJ11* (Kir6.2) and *ABCC8* (SUR1) were subcloned into pcDNA4/TO (for cDNA expression). The canine cDNAs were cloned from full-length first-strand cDNA (Dog Universal Reference cDNA, Zyagen), synthesized from universal reference total RNA (from a wide range of organs from both male and female beagle dogs). The human cDNAs were purchased from GenScript and codon-optimized for expression in HEK cells. Site-directed mutagenesis was performed using the QuikChange XL system (Stratagene, UK), and the resulting mutant clones were verified by sequencing (SourceBioScience, UK).

#### 2.1.1 HEK293T cells: maintenance, transfection and expression

HEK293T cells (SIGMA) were cultured in DMEM (4.5 g/L glucose; Sigma, UK) supplemented with 10% (vol./vol.) fetal bovine serum (FBS: Life Technologies, UK), 100U/ml penicillin, and 100 μg/ml streptomycin (ThermoFisher Scientific, UK) at 37 °C in a humidified atmosphere of 5% CO_2_/95% air. They were maintained at 50%–90% confluence. *Mycoplasma* contamination was negative when tested using MycoAlert *Mycoplasma* Detection Kit (Lonza Bioscience, UK).

For transfection, cells were seeded at 80% confluence in 25 cm^2^ flasks. After 24 h, they were transfected using TransIT-LT1 (Mirus Bio, USA) with 1.5 µg of wild-type or mutant Kir6.2 cDNA and 4.5 µg of wild-type SUR1 cDNA. Kir6.2 and SUR1 subunits were either both human, both canine or a combination (hybrid channels: human Kir6.2 + canine SUR1 [hKir6.2+cSUR1] or canine Kir6.2 + human SUR1 [cKir6.2+hSUR1]), as indicated in the Figures. K_ATP_ currents were recorded 24–48 h post transfection, after plating the cells onto 35 mm poly-L-lysine-coated Petri dishes (Corning, UK).

### 2.2 Electrophysiology

Macroscopic K_ATP_ currents were recorded from giant inside-out patches excised from transfected HEK293T cells. Data were acquired at −60 mV, using an Axopatch 200B amplifier (Molecular Devices), sampled at 10 kHz with a Digidata 1322A A/D-D/A converter, and controlled by pCLAMP9 software (Molecular Devices). Signals were filtered at 1 kHz.

The extracellular (pipette) solution contained (in mmol/l): 140 KCl, 1.2 MgCl_2_, 2.6 CaCl_2_ and 10 HEPES (pH 7.4, adjusted with KOH). The intracellular (bath) solution contained (in mmol/l): 107 KCl, 2 MgCl_2_, 1 CaCl_2_, 10 EGTA and 10 HEPES (pH 7.3 with KOH). The Mg^2+^-free intracellular solution comprised (in mmol/l): 140 KCl, 1 EDTA, 1 EGTA, 10 Hepes (pH 7.3, adjusted with KOH).

To control for K_ATP_ current rundown, nucleotide-containing test solutions were alternated with nucleotide-free solutions. Currents were normalized to the mean value in the control solution (I_C_) measured before and after each nucleotide application. ATP concentration-response curves were individually fitted with the following Hill equation:

$$\frac{I}{I_{c}}=\frac{1}{{1+\left(\frac{\left[ATP\right]}{{IC}_{50}}\right)}^{h}}$$

where *[ATP]* indicates the nucleotide concentration, *IC*_*50*_ is the concentration causing half-maximal inhibition, *I*_*C*_ is the current in control solution, *I* is the current in the presence of ATP, and *h* is the Hill coefficient. The ability of MgADP to stimulate channel activity was tested both in the presence and absence of MgATP.

### 2.3 Statistical analysis

When comparing two groups with a normal distribution and comparable variance, significant differences were calculated using a two-tailed, unpaired t-test. For multiple groups, significant differences were calculated with a one-way ANOVA with Sidak correction. All data are presented as mean ± SEM. N values are noted in each figure legend.

## 3 Results

### 3.1 Multi-species alignments of KCNJ11 gene

**FIGURE 1:**
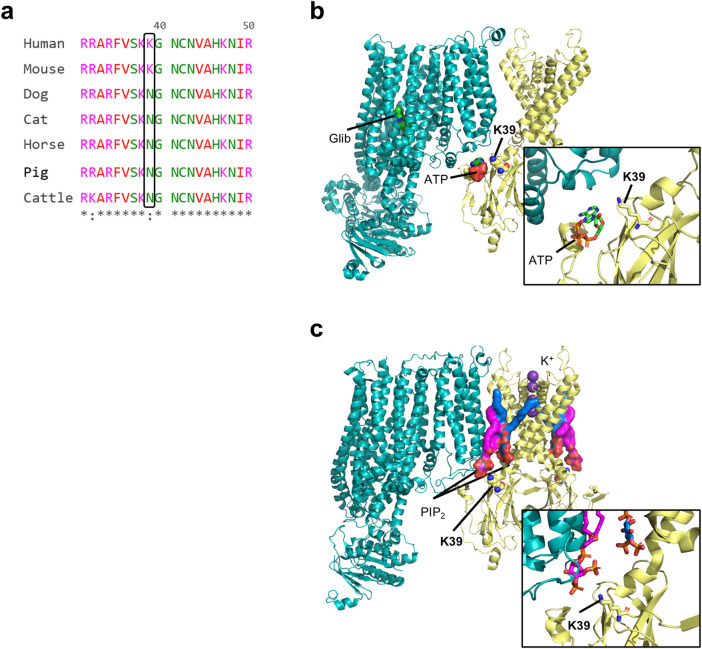
Multi-species protein sequence alignment of the Kir6.2 protein, encoded by the KCNJ11 gene and structure. **(a)** Portion of a multi-species sequence alignment for Kir6.2, highlighting key residue 39. Prepared with The EMBL-EBI Job Dispatcher sequence analysis tools framework in 2024. **(b,c)** Structural models of a closed rat wild-type Kir6.2/SUR1 K_ATP_ channel in complex with ATP and glibenclamide (b; PDB ID 6BAA), and of rat Kir6.2^Q52R^/SUR1 channel in complex with PIP_2_ (c; PDB ID 8T11). SUR is shown in teal, and Kir6.2 is shown in yellow. The two PIP_2_ are shown in magenta (non canonical site) and blue (canonical site). Only one SUR1 subunit and two Kir6.2 subunits are shown for clarity.

We performed a multi-species (human, mouse, dog, cat, horse, pig and cattle) sequence alignment of the Kir6.2 protein, encoded by the *KCNJ11* gene. The alignment revealed a very high degree of conservation (Figure 1; Supplementary Figure S1), particularly between the human, mouse and dog Kir6.2 sequences (Supplementary Figure S2). Notably, only two residues that are shared between human and mouse Kir6.2 are different in the canine sequence - K39N and L191V. Of these, residue 191 shows mixed conservation, with leucine (L) present in four out of seven species aligned. In contrast, position 39 a lysine (K) in human and mouse, but appears as an asparagine (N) in all other species examined. The K39N variant is of special significance as variation at this residue has been associated with ND. Residue 39 has previously been identified as an amino acid shared between the inhibitory ATP-binding pocket and the stimulatory PIP_2_-binding site (Figure 1b). Mutations at this residue influence the channel ATP sensitivity (Driggers et al., 2024; Pipatpolkai et al., 2022). We therefore investigated whether the human and canine K_ATP_ channels exhibit different ATP sensitivities due to variation of the K39N residue.

### 3.2 The ATP sensitivity of the canine K_ATP_ channel differs from that of the human channel

**FIGURE 2:**
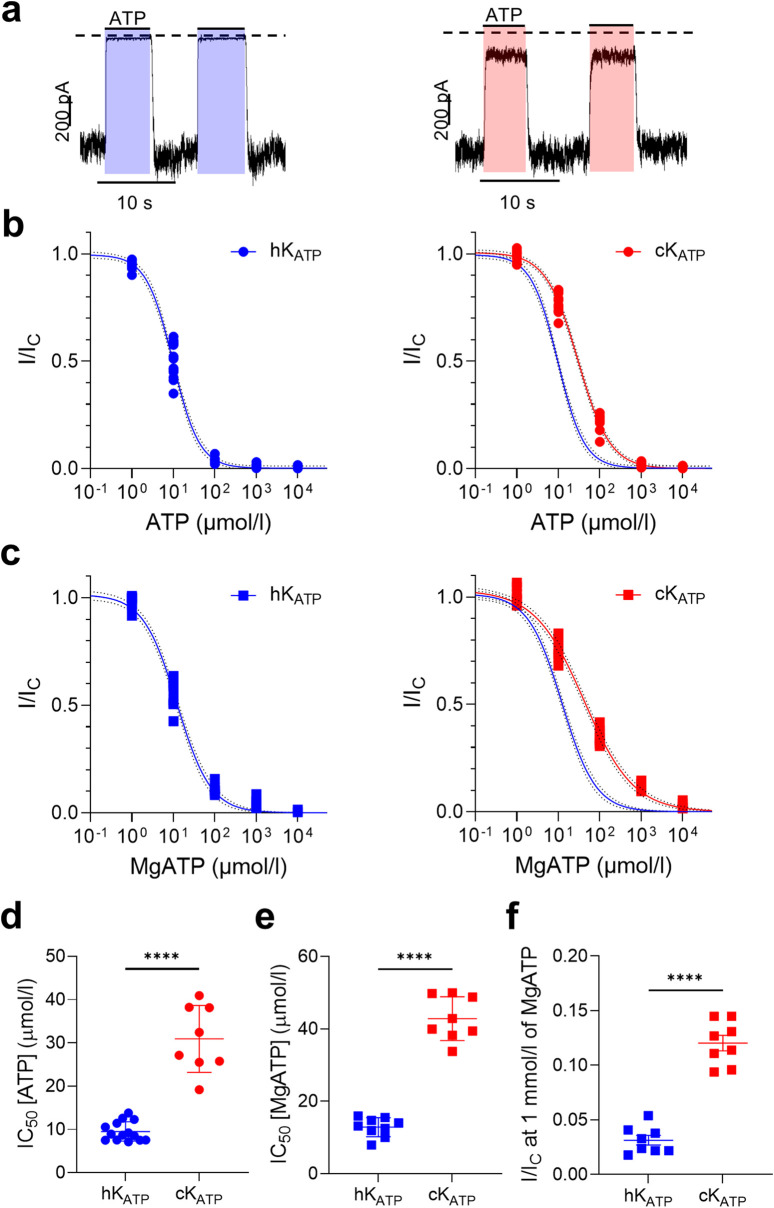
K_ATP_ current inhibition varies between human and dog. **(a)** Representative current traces for human (left) and canine (right) wild-type K_ATP_ channels recorded at −60 mV from inside-out patches excised from HEK293T cells exposed to 100 μmol/l ATP concentrations (indicated by the bars). The zero-current level is shown by the dashed lines. **(b,c)** ATP concentration-response curves in the absence (circle, **b**) and presence (squares, **c**) of Mg^2+^ for human (hK_ATP_: blue), and canine (cK_ATP_: red) channels. Current amplitudes (I) are expressed as a fraction of the maximum K_ATP_ current measured in control solution in the same patch (i.e., in the absence of ATP; Ic). The lines are the best fit of the Hill equation to the mean data. **(d,e)** Bar plots showing the corresponding IC_50_ values for each individual concentration-response curve, in the absence (circle, **d**) and presence (square, **e**) of Mg^2+^. **(f)** Residual current at 1 mmol/l MgATP. ****p < 0.0001, unpaired two-tail t-test; n = 8–13.

We expressed human or canine Kir6.2 with their respective species-specific SUR1 subunits in HEK293T cells and recorded K_ATP_ currents from inside-out membrane patches. ATP sensitivity was assessed by applying increasing concentrations of ATP to the intracellular side of the membrane and quantifying K_ATP_ current inhibition (Figure 2a). Comparison of the ATP concentration–response curves revealed a clear canine-human species difference: in the absence of Mg^2+^, the IC_50_ for ATP inhibition was ∼10 µM for the human K_ATP_ channel compared with ∼30 µM for the canine channel (Figures 2b,d; Supplementary Table S1). Likewise, the canine K_ATP_ channel exhibited a significantly reduced sensitivity to MgATP (Figures 2c,e; Supplementary Table S2) compared to human one. Additionally, the residual channel activity at 1 mmol/L ATP was significantly higher in canine channels (12.0% ± 0.7%, n = 8) than in human channels (3.2% ± 0.4%, n = 9) (Figure 2f).

### 3.3 MgADP stimulation does not vary between canine and human K_ATP_ channels

**FIGURE 3:**
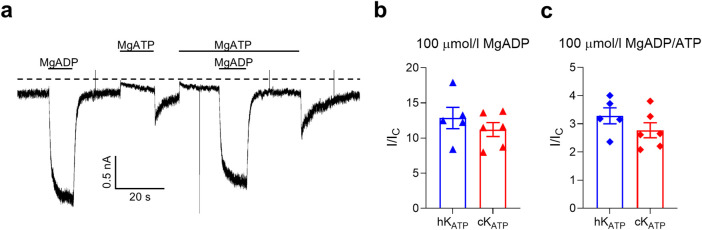
K_ATP_ current activation. **(a)** Representative current traces for canine wild-type K_ATP_ channels recorded at −60 mV from inside-out patches excised from HEK293T cells exposed to 100 μmol/l MgATP and/or 100 μmol/l MgADP (as indicated by the bars). The zero-current current level is shown by the dashed lines. **(b,c)** Bar plots showing the ADP-dependent fold increase of the K_ATP_ current amplitudes (compared to the current in control solution, Ic) for human (blue, n = 5) and canine (red, n = 6) channels. Individual data points and mean ± SEM are shown. There was no significant difference between the two groups when compared using an unpaired two-tail t-test.

We next examined whether the ability of MgADP to stimulate K_ATP_ channel activity differed between the two species. To do this, we applied 100 μmol/L MgADP either alone or in combination with 100 μmol/L MgATP and measured the resulting changes in K_ATP_ current. In both conditions, MgADP produced a comparable fold-increase in current amplitude in human and canine channels (Figure 3; Supplementary Table S3).

### 3.4 The Kir6.2 subunit is the primary determinant of ATP sensitivity

**FIGURE 4:**
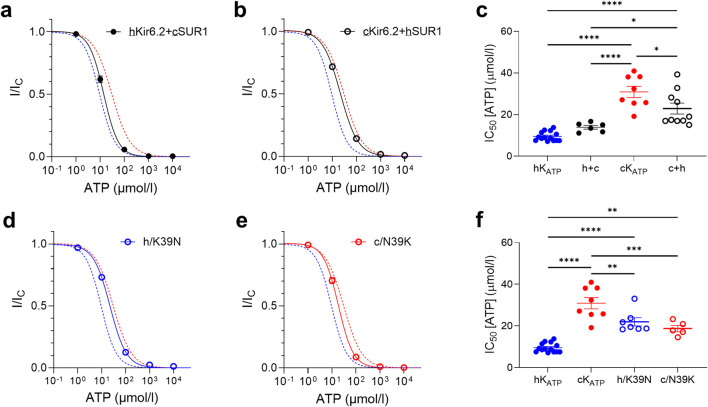
ATP sensitivity is mainly dictated by the Kir6.2 subunit. **(a,b)** ATP concentration-response curves and corresponding IC_50_ values **(c)** for hybrid channels (black) compared to the best Hill fit of human (blue dashed line) and canine (red dashed line) wild-type K_ATP_ curves. **(d,e)** ATP concentration-response curves and corresponding IC_50_ values **(f)**, for human (blue) and canine (red) K_ATP_ channels with Kir6.2 residue 39 swapped between them, in the absence of Mg^2+^. Wild-type curves are plotted for comparison. Mean currents (±SEM) are shown for each construct compared to the best Hill fit of human and canine wild-type K_ATP_ curves. *p < 0.05, **p < 0.01, ***p < 0.001, ****p < 0.0001, ordinary one-way ANOVA with Sidak correction, n = 5–13.

To dissect the relative contributions of the Kir6.2 and SUR1 subunits to the channel ATP sensitivity between species, we generated hybrid K_ATP_ channels by co-expressing human Kir6.2 with canine SUR1 (hKir6.2+cSUR1) and canine Kir6.2 with human SUR1 (cKir6.2+hSUR1). Electrophysiological recordings revealed that the ATP sensitivity of these hybrid channels closely followed that of the species origin of the Kir6.2 subunit, rather than that of the SUR1 subunit (Figures 4a,b). Specifically, the ATP sensitivity of the hKir6.2+cSUR1 channels was not significantly different from that of the human channel, whereas cKir6.2+hSUR1 channels exhibited an ATP sensitivity more closely resembling that of the canine channel.

To further elucidate the molecular basis of this difference, we introduced point mutations at residue 39 of Kir6.2 (Figure 1). Substitution of residue 39 in human Kir6.2 with the canine (N) residue at this position led to a significant reduction in ATP sensitivity, similar to that observed with the full canine Kir6.2 sequence. Conversely, substitution of residue 39 in canine Kir6.2 with the human (K) residue led to a significant restoration of ATP sensitivity, bringing the channel ATP sensitivity profile closer to that observed in human K_ATP_ channels (Figures 4d,e). This suggests that residue 39 plays a modulatory role in the channel’s response to ATP and may contribute to interspecies variation in K_ATP_ channel regulation.

## 4 Discussion

Our results show that the *KCNJ11* gene is broadly conserved across mammals, particularly at residues previously associated with important functions. However, residue 39, known to be important in ATP sensitivity, is a lysine in the human and mouse, but an asparagine in dog, cat, horse, pig, and cow. It is also the only other residue that is shared between the human and mouse *KCNJ11* gene, yet differs in dog.

Functional studies revealed that the canine channel is somewhat less sensitive to ATP than the human one, and that this difference is conferred by the Kir6.2 subunit rather than the SUR1 subunit. Furthermore, the K39N mutation in the human channel reduced K_ATP_ channel ATP sensitivity, whereas the N39K mutation in the canine channel increased the ATP sensitivity. This indicates that the difference in ATP sensitivity between the channels of the two species is conferred largely by the residue at position 39.

Atomistic molecular dynamic simulations and recent K_ATP_ channel structures suggest that K39 potentially competitively coordinates both the ATP and PIP_2_ binding sites (Figure 1) (Driggers et al., 2024; Pipatpolkai et al., 2022; Sung et al., 2022). In the ATP-bound closed structure, ATP interacts with K39; conversely in the PIP_2_-bound open structure, the side-chain of K39 no longer stabilises ATP binding, but instead flips away and interacts with the head group of PIP_2_ at the non-canonical site (Driggers et al., 2024). This accounts for the antagonism between ATP and PIP_2_ which respectively inhibit and stimulate channel activity (Baukrowitz et al., 1998; Shyng and Nichols, 1998; Driggers et al., 2024) and explains why the human Kir6.2-K39R mutation reduces ATP sensitivity and causes neonatal diabetes, and similarly explains why the canine channel is less ATP-sensitive than the human channel.

### 4.1 Clinical implications

The difference in ATP sensitivity between the dog and human channel is small but significant: a shift of ∼20 μmol/L in the IC_50_ for ATP and of ∼30 μmol/L for MgATP (Supplementary Tables S1, S2). The current at 1 mmol/L MgATP is also 4-fold greater for the canine channel. It is plausible that this difference may translate to differences in glucose homeostasis between the two species.

Although the difference in ATP sensitivity between canine and human channels is relatively modest, even small shifts in current can have pronounced effects on membrane potential and insulin release, especially near the threshold, due to the high input resistance of the beta-cell membrane. For example, the E23K variant in human *KCNJ11*, which causes an even smaller shift in ATP sensitivity than that observed here, is associated with an increased risk of T2D (Gloyn et al., 2003). Mice carrying the K variant at residue 23 also show impaired glucose-stimulated insulin secretion, particularly at threshold levels of glucose (5–7 mmol/L) (Sachse et al., 2021). Mutations in the human channel that cause small reductions in ATP sensitivity give rise to young onset diabetes or transient or permanent neonatal diabetes (D'Amato et al., 2008; Vedovato et al., 2016). These findings suggest that canine K_ATP_ channels might remain open at glucose concentrations that normally would trigger insulin release in humans, implying differences in canine glucose homeostasis and potentially impacting diabetes susceptibility in the canine species.

## 5 Conclusion

Our findings demonstrate that the *KCNJ11* gene is broadly conserved across mammals, yet specific variations in the Kir6.2 subunit contribute to differences in the ATP sensitivity of the beta-cell K_ATP_ channel. Specifically, canine K_ATP_ channels exhibit reduced ATP-mediated inhibition compared to human channels, a difference that appears to map primarily to the Kir6.2 subunit, rather than SUR1.

Mutation analysis at residue 39 partially recapitulated this species-associated phenotype, supporting its modulatory role in ATP sensitivity. In contrast, MgADP stimulation was conserved between human and canine channels, indicating that ATP sensitivity is the primary divergent feature.

While the physiological implications for insulin secretion and beta-cell function remain to be fully understood, these results underscore the importance of considering species-specific molecular differences when investigating K_ATP_ channel regulation.

## Data Availability

The raw data supporting the conclusions of this article will be made available by the authors, without undue reservation.

## Abbreviations

ATP, adenosine triphosphate; ADP, adenosine diphosphate; K_ATP_, ATP-sensitive potassium channel; Kir6.2, inwardly rectifying potassium channel; ND, neonatal diabetes; PIP_2_, phosphatidylinositol 4,5-bisphosphate; SUR1, regulatory sulfonylurea receptor 1.
