# Supplementary material for: Residue 39 of Kir6.2 drives a difference in ATP sensitivity in human and canine beta-cell KATP channels
Source: Front Physiol. 2025 Oct 17;16:1693112. doi: 10.3389/fphys.2025.1693112 (PMC12580652; doi:10.3389/fphys.2025.1693112)
Supplement: Supplementary file 1 [file DataSheet1.pdf]

Supplement Figure 1

```
Human     MLSRKGIIPEEYVLTRLAEDPAKPRYRARQRRARFVSKKGNCNVAHKNIREQGRFLQDVF    60
Mouse     MLSRKGIIPEEYVLTRLAEDPAEPRYRTRERRARFVSKKGNCNVAHKNIREQGRFLQDVF    60
Dog       MLSRKGIIPEEYVLTRLAEDPAEPRYRARERRARFVSKNGNCNVAHKNIREQGRFLQDVF    60
Cat       MLSRKGIIPEEYVLTRLAEDPAEPRYRARERRARFVSKNGNCNVAHKNIREQGRFLQDVF    60
Horse     MLSRKGIIPEEYVLTRLAEDPAEPRYRARERRARFVSKNGNCNVAHKNIREQGRFLQDVF    60
Pig       MLSRKGIIPEEYVLTRLAEDPTEPRYRARERRARFVSKNGNCNVAHKNIREQGRFLQDVF    60
Cattle    MLSRKGIIPEEYVLTRLAEDPAEPRYRARERKARFVSKNGNCNVAHKNIREQGRFLQDVF    60
          *********************::****:*:*:******:*********************

Human     TTLVDLKWPHTLLIFTMSFLCSWLLFAMAWWLIAFAHGDLAPSEGTAEPCVTSIHSFSSA    120
Mouse     TTLVDLKWPHTLLIFTMSFLCSWLLFAMVWWLIAFAHGDLAPGEGTNVPCVTSIHSFSSA    120
Dog       TTLVDLKWPHTLLIFTMSFLCSWLLFAMVWWLIAFAHGDLAPGEGTAVPCVTSIHSFSSA    120
Cat       TTLVDLKWPHTLLIFTMSFLCSWLLFAMVWWLIAFAHGDLAPGEGAAVPCVTSIHSFSSA    120
Horse     TTLVDLKWPHTLLIFTMSFLCSWLLFAMAWWLIAFAHGDLAPDEGSAVPCVTSIHSFSSA    120
Pig       TTLVDLKWPYTLLIFTMSFLCSWLLFAMVWWLIAFAHGDLAPGEGASVPCVTSIHSFSSA    120
Cattle    TTLVDLKWPYTLLIFTMSFLCSWLLFAMVWWLIAFAHGDLAPGEGAAVPCVTSIHSFSSA    120
          *********:******************.*************.**:  ************

Human     FLFSIEVQVTIGFGGRMVTEECPLAILILIVQNIVGLMINAIMLGCIFMKTAQAHRRAET    180
Mouse     FLFSIEVQVTIGFGGRMVTEECPLAILILIVQNIVGLMINAIMLGCIFMKTAQAHRRAET    180
Dog       FLFSIEVQVTIGFGGRMVTEECPLAILILIVQNIVGLMINAIMLGCIFMKTAQAHRRAET    180
Cat       FLFSIEVQVTIGFGGRMVTEECPLAILILIVQNIVGLMINAIMLGCIFMKTAQAHRRAET    180
Horse     FLFSIEVQVTIGFGGRMVTEECPLAILILIVQNIVGLMINAIMLGCIFMKTAQAHRRAET    180
Pig       FLFSIEVQVTIGFGGRMVTEECPLAILILIVQNIVGLMINAIMLGCIFMKTAQAHRRAET    180
Cattle    FLFSIEVQVTIGFGGRMVTEECPLAILILIVQNIVGLMINAIMLGCIFMKTAQAHRRAET    180
          ************************************************************

Human     LIFSKHAVIALRHGRLCFMLRVGDLRKSMIISATIHMQVVRKTTSPEGEVVPLHQVDIPM    240
Mouse     LIFSKHAVITLRHGRLCFMLRVGDLRKSMIISATIHMQVVRKTTSPEGEVVPLHQVDIPM    240
Dog       LIFSKHAVIAVRHGRLCFMLRVGDLRKSMIISATIHMQVVRKTTSPEGEVVPLHQVDIPM    240
Cat       LIFSKHAVIAVRHGRLCFMLRVGDLRKSMIISATIHMQVVRKTTSPEGEVVPLHQVDIPM    240
Horse     LIFSKHAVIALRHSRLCFMLRVGDLRKSMIISATIRMQVVRKTTSPEGEVVPLHQVEIPM    240
Pig       LIFSKHAVIAVRHGRLCFMLRVGDLRKSMIISATIHMQVVRKTTSPEGEVVPLHQVDIPM    240
Cattle    LIFSKHAVIALRHGRLCFMLRVGDLRKSMIISATIHMQVVRKTTSPEGEVVPLHQVDIPM    240
          *********::**.*********************:********************:***

Human     ENGVGGNSIFLVAPLIIYHVIDANSPLYDLAPSDLHHHQDLEIIVILEGVVETTGITTQA    300
Mouse     ENGVGGNGIFLVAPLIIYHVIDSNSPLYDLAPSDLHHHQDLEIIVILEGVVETTGITTQA    300
Dog       ENGVGGNSIFLVAPLIIYHVIDANSPLYDLAPSDLHHHQDLEIIVILEGVVETTGITTQA    300
Cat       ENGVGGNSIFLVAPLIIYHVIDANSPLYDLAPSDLHHHQDLEIIVILEGVVETTGITTQA    300
Horse     ENGVGGNSIFLVAPLIIYHAIDANSPLYDLAPSDLHHHQDLEIIVILEGVVETTGITTQA    300
Pig       ENGVGGNNIFLVAPLIIYHIIDAKSPLYDLAPCDLHHHQDLEIIVILEGVVETTGITTQA    300
Cattle    ENGVGGNSIFLVAPLIIYHVIDANSPLYDLAPCDLHHHQDLEIIVILEGVVETTGITTQA    300
          *******.*********** **::********.***************************

Human     RTSYLADEILWGQRFVPIVAEEDGRYSVDYSKFGNTVKVPTPLCTARQLDEDHSLLEALT    360
Mouse     RTSYLADEILWGQRFVPIVAEEDGRYSVDYSKFGNTIKVPTPLCTARQLDEDRSLLDALT    360
Dog       RTSYLADEILWGQRFVPIVAEEDGRYSVDYSKFGNTIKVPTPLCTARQLDEDRSLLDALT    360
Cat       RTSYLADEILWGQRFVPIVAEEDGRYSVDYSKFGNTIKVPTPLCTARQLDEDRTLLDAVT    360
Horse     RTSYLADEILWGQRFVPIVAEEDGRYSVDYSKFGNTIKVPTPLCTARQLDEDHSLLDALT    360
Pig       RTSYLADEILWGQRFVPIVAEEDGRYTVDYSKFGNTIKVPTPLCSARQLDEDPSLLDVLT    360
Cattle    RTSYLADEILWGQRFVPIVAEEDGRYSVDYSKFGNTIKVPTPLCTARQLEEDPSLLDVLT    360
          **************************:*********:*******:****:** :**:.:*

Human     LASARGPLRKRSVPMAKAKPKFSISPDSLS  390
Mouse     LASSRGPLRKRSVAVAKAKPKFSISPDSLS  390
Dog       LASARGPLRKRSVAVAKAKPKFSISPDSLS  390
Cat       LASARGPLRKRSVAVAKAKPKFSISPDSLS  390
Horse     LASARGPLRKRSVAVAKAKPKFSISPDSLS  390
Pig       L--TRGPLRKRSMAVTKAKPKFSISPESLS  388
Cattle    L--VRGPLRKRTVAVAKAKPKFSISPDSLS  388
          *   *******:: ::**********:***
```

Multi-species protein sequence alignment of the Kir6.2 protein, encoded by the *KCNJ11* gene. Full multi-species sequence alignment for Kir6.2, for the same species shown in figure 1. Prepared using "The EMBL-EBI Job Dispatcher sequence analysis tools framework in 2024".

Supplement Figure 2

```
Human    MLSRKGIIPEEYVLTRLAEDPAEPRYRARQRRARFVSKKGNCNVAHKNIREQGRFLQDVF    60
Mouse    MLSRKGIIPEEYVLTRLAEDPAEPRYRTRERRARFVSKKGNCNVAHKNIREQGRFLQDVF    60
Dog      MLSRKGIIPEEYVLTRLAEDPAEPRYRARERRARFVSKNGNCNVAHKNIREQGRFLQDVF    60
         ***************************:*:********:*********************

Human    TTLVDLKWPHTLLIFTMSFLCSWLLFAMAWWLIAFAHGDLAPSEGTAEPCVTSIHSFSSA    120
Mouse    TTLVDLKWPHTLLIFTMSFLCSWLLFAMVWWLIAFAHGDLAPGEGTNVPCVTSIHSFSSA    120
Dog      TTLVDLKWPHTLLIFTMSFLCSWLLFAMVWWLIAFAHGDLAPGEGTAVPCVTSIHSFSSA    120
         ****************************.*************.***  ************

Human    FLFSIEVQVTIGFGGRMVTEECPLAILILIVQNIVGLMINAIMLGCIFMKTAQAHRRAET    180
Mouse    FLFSIEVQVTIGFGGRMVTEECPLAILILIVQNIVGLMINAIMLGCIFMKTAQAHRRAET    180
Dog      FLFSIEVQVTIGFGGRMVTEECPLAILILIVQNIVGLMINAIMLGCIFMKTAQAHRRAET    180
         ************************************************************

Human    LIFSKHAVIALRHGRLCFMLRVGDLRKSMIISATIHMQVVRKTTSPEGEVVPLHQVDIPM    240
Mouse    LIFSKHAVITLRHGRLCFMLRVGDLRKSMIISATIHMQVVRKTTSPEGEVVPLHQVDIPM    240
Dog      LIFSKHAVIAVRHGRLCFMLRVGDLRKSMIISATIHMQVVRKTTSPEGEVVPLHQVDIPM    240
         *********::*************************************************

Human    ENGVGGNSIFLVAPLIIYHVIDANSPLYDLAPSDLHHHQDLEIIVILEGVVETTGITTQA    300
Mouse    ENGVGGNGIFLVAPLIIYHVIDSNSPLYDLAPSDLHHHQDLEIIVILEGVVETTGITTQA    300
Dog      ENGVGGNSIFLVAPLIIYHVIDANSPLYDLAPSDLHHHQDLEIIVILEGVVETTGITTQA    300
         *******.**************:*************************************

Human    RTSYLADEILWGQRFVPIVAEEDGRYSVDYSKFGNTIKVPTPLCTARQLDEDHSLLEALT    360
Mouse    RTSYLADEILWGQRFVPIVAEEDGRYSVDYSKFGNTIKVPTPLCTARQLDEDRSLLDALT    360
Dog      RTSYLADEILWGQRFVPIVAEEDGRYSVDYSKFGNTIKVPTPLCTARQLDEDRSLLDALT    360
         ****************************************************:***:***

Human    LASARGPLRKRSVPMAKAKPKFSISPDSLS  390
Mouse    LASSRGPLRKRSVAVAKAKPKFSISPDSLS  390
Dog      LASARGPLRKRSVAVAKAKPKFSISPDSLS  390
         ***:********* :***************
```

Multi-species protein sequence alignment of the Kir6.2 protein, encoded by the *KCNJ11* gene. Full sequence alignment of human, mouse and canine Kir6.2. Most residues are conserved among the three species; furthermore, only two divergent residues are shared between the human and mouse sequences but differ in the canine sequence. Of these, only residue 39 (lysine in humans) is in a location predicted to be functionally significant. The alignment was generated using the “EMBL-EBI Job Dispatcher sequence analysis tools (2024)”.

Supplementary tables

Table 1. Mean ± SEM from Hill equation fits to individual ATP dose-responses for data in figures 1,3.

ATP.K_2	hK_{ATP} (n=13)	cK_{ATP} (n=8)	hKir6.2 +cSUR1 (n=6)	cKir6.2 +hSUR1 (n=10)	hK_{ATP}/K39N (n=7)	cK_{ATP}/N39K (n=5)
IC_{50} (µmol/L)	9.7±0.6	31±3	14±1	23±3	22±2	19±1
h	1.44±0.05	1.19±0.04	1.51±0.12	1.29±0.05	1.30±0.03	1.43±0.05

Table 2. Mean ± SEM from Hill equation fits to individual MgATP dose-responses and residual currents at 1mmol/L and 3mmol/L MgATP for data in figures 1,3. Data for 3 mmol/L are extrapolated.

MgATP	hK_{ATP} (n=9)	cK_{ATP} (n=8)
IC_{50} (µmol/L)	13±1	43±2
h	1.12±0.05	0.81±0.05
I/I_C (%) (1mmol/L)	3.2±0.4	12.0±0.7
I/I_C (%) (3mmol/L)	0.36±0.07	5.8±0.7

Table 3. Mean ± SEM of the current fold change for MgADP activation shown in figure 2.

I/I_C	hK_{ATP} (n=5)	cK_{ATP} (n=6)
MgADP (100 µmol/L)	13±2	11±1
MgADP/ATP (100 µmol/L each)	3.3±0.3	2.8±0.3
